# Group A Streptococcal Septic Arthritis and Osteomyelitis Presenting As Acute Rheumatic Fever: A Diagnostic Dilemma

**DOI:** 10.7759/cureus.117456

**Published:** 2026-10-04

**Authors:** Ethan M Gardner, Tara L Greenhow

**Affiliations:** 1 Research, Kaiser Permanente Northern California, Pleasanton, USA; 2 Pediatrics, Kaiser Permanente Northern California, San Francisco, USA; 3 Advancing Research in Children's Health (ARCH) Network, Kaiser Permanente Northern California, Pleasanton, USA

**Keywords:** acute rheumatic fever, group a streptococcus, osteomyelitis, poststreptococcal reactive arthritis, septic arthritis

## Abstract

Historically, it has been difficult to differentiate between acute rheumatic fever (ARF) and poststreptococcal reactive arthritis (PSRA), given their overlapping systemic symptoms and antecedent *Streptococcus pyogenes* (Group A Streptococcus (GAS)) diagnosis. We report the case of a five-year-old girl who presented with fever, chills, body aches, and swollen joints. A positive throat qualitative polymerase chain reaction (PCR) test for GAS and significant right knee and bilateral elbow pain prompted admission. The child met the Jones diagnostic criteria for ARF; however, arthrocentesis and imaging later confirmed a septic left elbow and adjacent radial and olecranon osteomyelitis. In the context of this finding, and given the patient’s additive, non-suppurative polyarthritis in other afflicted joints, it was deemed more likely that the patient had simultaneous PSRA and septic arthritis rather than ARF. Our case demonstrates how concurrent infection can misrepresent clinical indicators under the Jones criteria, emphasizing the need for a nuanced, risk-averse approach to managing poststreptococcal complications.

## Introduction

Acute rheumatic fever (ARF) is a non-suppurative autoimmune response to Group A Streptococcus (GAS), manifesting in multiple organ systems with a poor prognosis if untreated [1]. It is strongly linked to the development of inflammatory cardiac complications, Sydenham's chorea, and rheumatic heart disease (RHD) [2]. Use of the 2015 revised Jones criteria is the standard practice for identifying ARF, wherein the presence of two major criteria (carditis, arthritis, chorea, erythema marginatum, subcutaneous nodules), or one major and two minor criteria (fever, arthralgia, prolonged PR interval, and elevated inflammatory markers including C-reactive protein (CRP) and erythrocyte sedimentation rate (ESR)), is sufficient to diagnose a first episode [3]. While the Jones criteria have undergone several revisions for accuracy, the lack of conclusive laboratory or clinical metrics makes definitive recognition of ARF fundamentally difficult [4]. Furthermore, there is diagnostic overlap with poststreptococcal reactive arthritis (PSRA), which is similarly a sequela of GAS infection and has symptoms of acute arthritis [5,6]. PSRA does not fulfill the Jones criteria in isolation, although concurrent or unrelated conditions may contribute findings that result in apparent fulfillment of the criteria [6,7]. PSRA can be further differentiated from ARF in that it has a generally faster onset and longer duration to resolution, responds less favorably to nonsteroidal anti-inflammatory drugs (NSAIDs), and tends to present with additive arthritis rather than the typical migratory arthritis of ARF [8-10]. Nevertheless, the distinction between ARF and PSRA remains ambiguous, with some clinical perspectives arguing that PSRA is an early, mild presentation of ARF [9,10]. The Jones criteria can be a useful tool to differentiate between the two in ideal clinical settings; there is, however, a sparsity of literature discussing how concurrent pathologies may convolute the distinction in more complex cases.

## Case presentation

A five-year-old female was seen by her primary care physician with a chief complaint of fevers ranging up to 105.0˚F and body aches for two days. The child endorsed transient non-bloody, non-bilious emesis but denied other gastrointestinal distress. She was presumed to have influenza and was treated with oseltamivir. Fever, significant additive myalgias and arthralgias of the upper and lower extremities, and mild upper respiratory symptoms persisted, necessitating five subsequent clinical encounters.

Complete blood count (CBC) taken on day 12 demonstrated a white blood cell count (WBC) of 22,200/μL, hemoglobin (Hb) of 11.4 g/dL, CRP of 2.5 mg/dL, and ESR of 88 mm/h (Table 1). Blood cultures were obtained and were negative. The child noted that pain had migrated to the wrists, right knee, and ankles. There was no notable swelling, and she did not experience chest pain or irregular heart rate. Physical examination yielded no evidence of conjunctival infection, strawberry tongue, or dry lips. Pharyngeal erythema was not observed, nor did the child complain of throat pain.

**Table 1:** Timeline of laboratory parameters

Parameter	Unit	Day 12	Day 18	Day 20	Day 22	Day 38	Day 53	Reference Range
Hematology								
White blood cell	x10^3^/μL	22.2	18.8	14.3	6.2	17.8	10.2	4.5-15.5
Hemoglobin	g/dL	11.4	9.1	9.1	8.6	10.1	11.0	11.5-13.5
Hematocrit	%	35.0	26.7	26.7	26.7	31.1	33.3	34.0-40.0
Platelet	x10^3^/μL	796	745	832	595	709	363	140-400
Chemistry								
Alanine aminotransferase	U/L	-	7	-	-	-	-	0-41
Creatinine	mg/dL	-	<0.20	-	-	-	-	0.30-0.70
Inflammatory markers								
Erythrocyte sedimentation rate	mm/h	88	74	-	>140	98	36	0-20
C-reactive protein	mg/L	2.5	9.9	11.3	3.3	<0.5	<0.5	≤0.9
Microbiology								
Antistreptolysin O	U/mL	-	876	-	-	>2000	-	≤200
Anti-DNase B	U/mL	-	<86	-	-	<86	-	≤249

Recurrent fever prompted a multiplex polymerase chain reaction (PCR) respiratory virus panel and a qualitative PCR throat swab for GAS on day 14. Peribronchial cuffing was observed via chest X-ray, and the child was started on azithromycin under suspicion of atypical pneumonia. The qualitative PCR assay confirmed GAS infection on day 16, and the child was prescribed amoxicillin. Fevers resolved for two days after initiation of amoxicillin, then returned with persistent right knee and bilateral elbow pain and swelling, leading to hospital admission for suspected ARF 17 days after initial complaint.

At admission, the child notably refused to ambulate and had difficulty straightening and moving both knees and elbows, particularly the left elbow and right knee. There was also sensitivity of the right wrist, which had resolved by the second examination. Hips and ankles had full range of motion. While the arthritis and arthralgia were polyarticular in nature, the left elbow was markedly swollen and tender to palpation. Erythema marginatum, subcutaneous nodules, and choreiform movements were each absent. CBC demonstrated a WBC of 18,800/μL, Hb of 9.1 g/dL, CRP of 9.9 mg/dL, and ESR of 74 mm/h. An antistreptolysin O (ASO) titer returned elevated at 876 (Table 1). Blood culture was repeated and was negative. An electrocardiogram was obtained as part of the Jones criteria assessment and demonstrated a normal PR interval. A left elbow X-ray (Figure 1) and ultrasound confirmed an effusion and diffuse soft tissue swelling with internal debris. X-rays indicated no irregularities in the knees, and arthrocentesis of the right knee was attempted and failed. Aspiration and washout of the left elbow revealed a synovial WBC of 85,080/μL with 96% polymorphonuclear cells and 4% lymphocytes. Synovial Gram stain showed moderate WBC with no organism seen; cultures were negative, but broad-range PCR confirmed GAS. Intravenous cefazolin was started for treatment of presumed septic arthritis, as well as ketorolac. WBC normalized to 6,200/μL within four days following administration of the regimen, and CRP decreased to 3.3 mg/dL. The full timeline of laboratory parameters can be found in Table 1.

**Figure 1:**
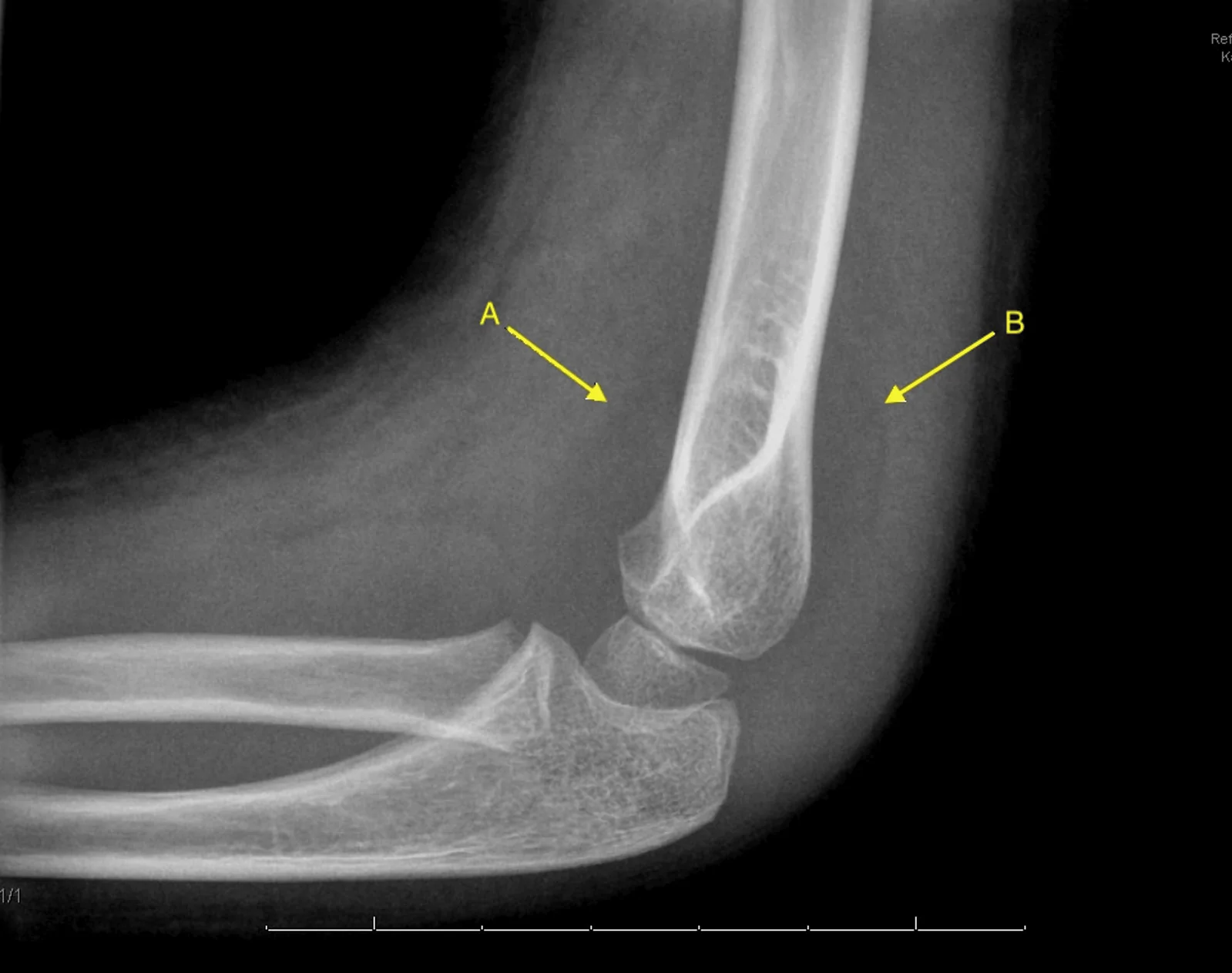
Lateral left elbow X-ray with large joint effusion (A) Raised anterior fat pad; (B) Visible posterior fat pad.

The child remained febrile with a limited range of motion and point tenderness of the left elbow. An echocardiogram was performed on hospital day 2, which found no irregularities. Magnetic resonance imaging of the left elbow was performed on hospital day 4, which demonstrated marked joint effusion with prominent synovitis most compatible with septic arthritis, as well as areas of bone marrow edema and enhancement within the olecranon and radial metaphysis indicative of osteomyelitis (Figures 2-3).

**Figure 2:**
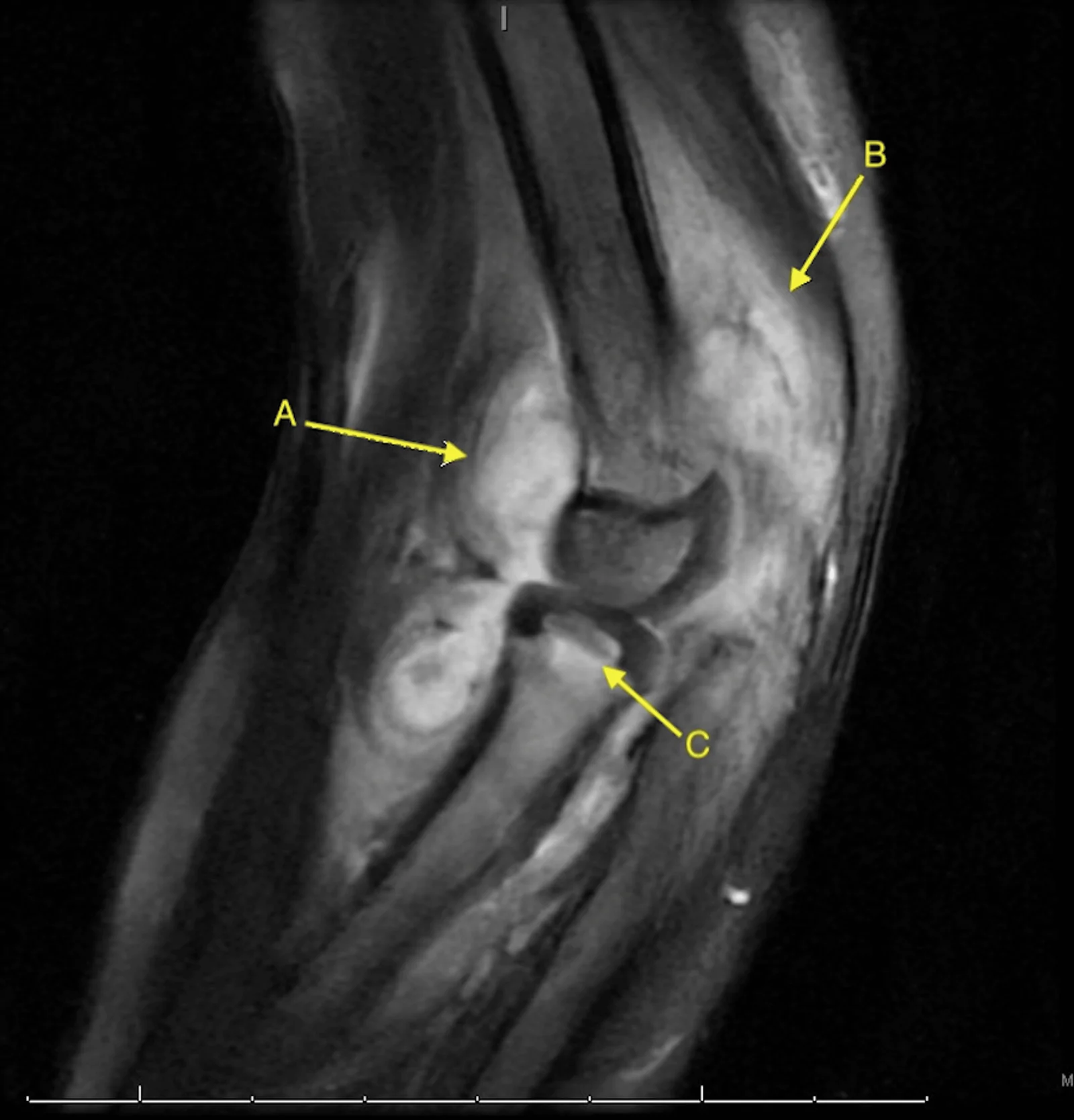
Sagittal T2-weighted fat-saturated magnetic resonance imaging of the left elbow with marked joint effusion and radial metaphyseal osteomyelitis (A) Anterior joint effusion; (B) Posterior joint effusion; (C) Edema and enhancement within the proximal radial metaphysis indicative of osteomyelitis.

**Figure 3:**
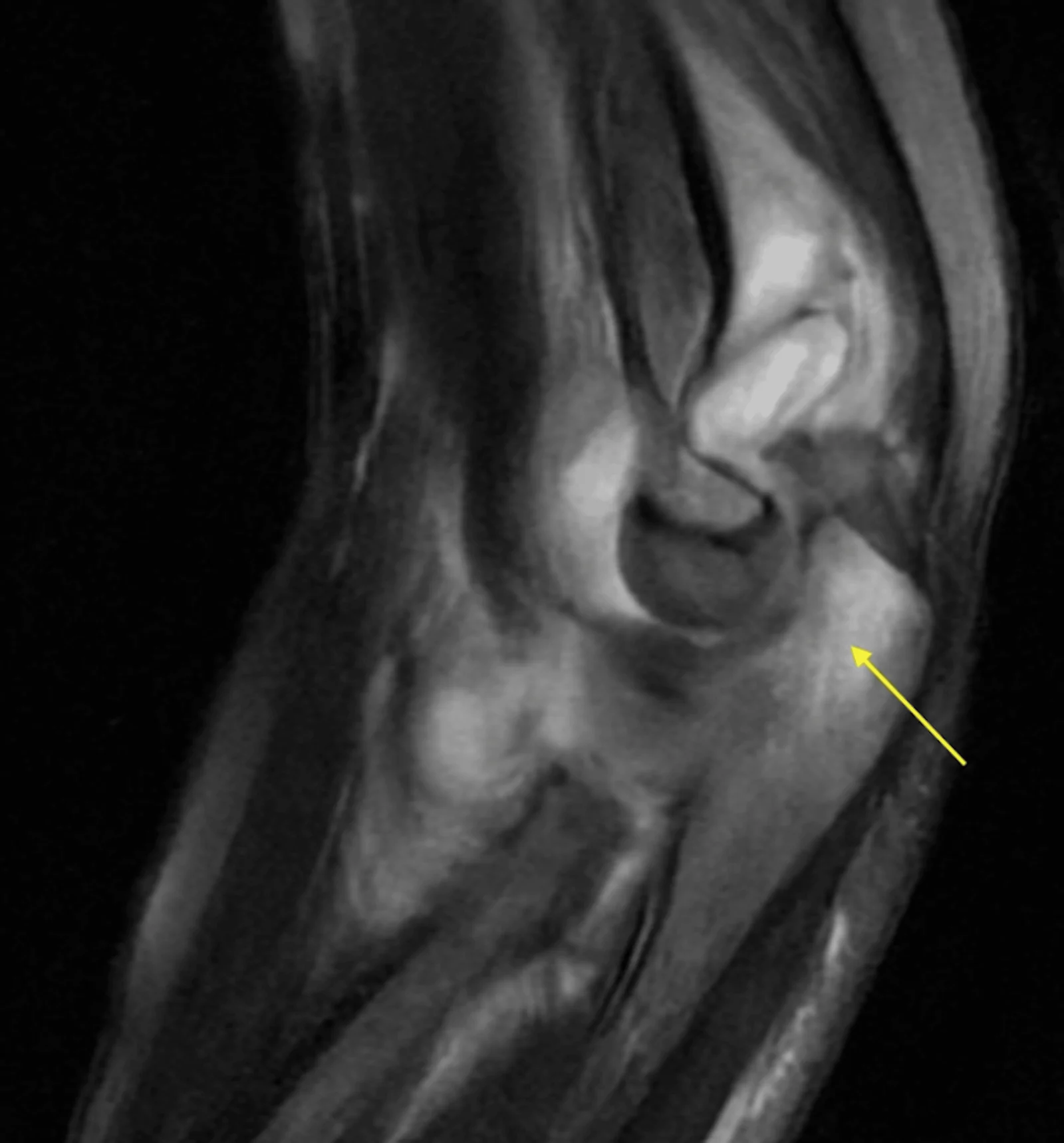
Sagittal T2-weighted fat-saturated magnetic resonance imaging of the left elbow demonstrating edema and enhancement within the olecranon metaphysis indicative of osteomyelitis (arrow)

The child was discharged eight days after admission on cephalexin to complete a six-week total antibiotic duration followed by one year of amoxicillin prophylaxis per American Heart Association (AHA) [10] and Canadian Infectious Diseases and Immunization Committee [9] guidelines for PSRA.

At outpatient follow-up one month after hospitalization, the pediatric infectious disease clinician found that all joint swelling had resolved and inflammatory markers were returning to normal. The child did not report feeling any pain, though her left elbow retained limited range of motion. She had returned to unrestricted physical activity.

## Discussion

Differential diagnosis

At initial assessment by the child’s primary care physician, the child’s high fevers, congestion, headache, and chills were sufficient to treat the case as influenza. However, persistent fever and progressive arthralgia after a full course of oseltamivir broadened the differential.

A rheumatologic evaluation including antinuclear antibody panel and rheumatoid factor was negative, as were infectious studies for Lyme disease and tuberculosis. There was no evidence of bacteremia, delaying consideration of a septic joint until arthrocentesis and PCR analysis of the left elbow were performed. Although the pediatric infectious disease team questioned whether all swollen joints could be infected with GAS, this was considered less likely than reactive arthritis due to the considerable distribution of arthritis and arthralgia, and that the majority of joint effusion had resolved by admission. A normal X-ray of the knees and the non-suppurative nature of the other afflicted joints supported this inference.

Because of the protracted emergence of the septic joint, the child was admitted with suspicion of ARF presenting with polyarthritis, fever, and elevated inflammatory markers. As more data became available, the pediatric infectious disease team determined that the left elbow was septic, and the diagnosis pivoted to septic joint and osteomyelitis with concurrent PSRA.

Treatment course

The child was treated empirically for influenza upon first examination. After a chest X-ray identified possible atypical pneumonia, the regimen transitioned to antibiotic therapy with azithromycin. Amoxicillin was added as GAS was identified via throat swab. Once septic arthritis with adjacent osteomyelitis was established, the treatment plan transitioned to intravenous antibiotics and NSAIDs. The patient received ibuprofen as the primary NSAID on the day of admission, followed by ketorolac for the next three days and then naproxen for the remainder of the hospital stay and as outpatient medication. Upon discharge, the patient was prescribed cephalexin to complete a six-week course. Given polyarticular arthralgia and arthritis, concurrent PSRA was felt likely, and the infectious disease team elected for one year of amoxicillin prophylaxis and an echocardiogram follow-up at completion.

Clinical assessment

The diagnosis of septic left elbow and adjacent radial and olecranon osteomyelitis with concurrent PSRA revealed itself late during the patient’s evaluation. This can be partially attributed to the initial empiric treatment for influenza, which delayed targeted antimicrobial therapy and likely facilitated the dissemination of the GAS infection. The diagnosis may have been considered earlier had the patient received a GAS swab at initial examination.

Once GAS was identified, the polyarthritis, persistent fever, and elevated acute-phase reactants were sufficient for the child to be admitted under suspicion of ARF. Consistent with ARF were the location of joint swelling (knees, ankles, wrists, and elbows), intermittent refusal to walk, the positive GAS swab, and elevated ASO titer. However, the differential diagnosis was obfuscated by the relatively static, additive rather than migratory polyarthritis, lack of resolution on NSAIDs, and onset while still symptomatic for GAS infection, each of which is indicative of PSRA.

The ambiguity introduced by the overlapping systemic symptoms of ARF and PSRA was exacerbated by the presence of a concurrent infection, delaying accurate diagnosis. The septic left elbow and adjacent radial and olecranon osteomyelitis triggered an inflammatory response, providing an alternative explanation for the immunological qualifiers under the Jones criteria.

The identification of septic arthritis and osteomyelitis was pivotal in distinguishing between ARF and PSRA in this case. The diagnosis of septic arthritis was highly suspected with the markedly abnormal synovial fluid analysis and confirmed by the positive broad-range PCR [11,12]. ARF and PSRA are non-suppurative complications of GAS infection and are by definition mutually exclusive from septic arthritis [13]. As such, once septic arthritis was recognized, the elevated inflammatory markers previously considered as minor Jones criteria were more appropriately attributed to the suppurative joint and bone infection rather than to a post-streptococcal inflammatory process. Without sufficient fulfillment of the Jones criteria, the diagnosis shifted from ARF to PSRA, which is more broadly defined as inflammatory arthritis occurring in the setting of GAS infection [14]. Given the additive polyarthralgia present apart from the septic arthritis, PSRA was maintained as part of the differential diagnosis. The patient was therefore treated for septic arthritis of the left elbow with adjacent osteomyelitis and concurrent PSRA.

The incidence of PSRA is one to two cases per 100,000 persons in the United States, which is nearly double that of ARF [15]. Despite its higher prevalence, the clinical presentation of PSRA is variable and notorious for mimicking other joint pathologies [16,17]. Notably, PSRA can manifest as clinically indistinguishable from acute septic arthritis, with negative synovial fluid cultures serving as the primary discriminator [17]. This is because the joint inflammation in PSRA is driven by immunologic cross-reactivity rather than localized bacterial invasion [18]. However, synovial fluid cultures in septic arthritis have also been found to be sterile in up to 35% of cases, impeding accurate differentiation [19]. In our case, the child did have negative cultures, which was explained by antibiotic pre-treatment; however, the elevated synovial fluid WBC counts, positive GAS synovial fluid PCR, and adjacent osteomyelitis were adequate to confirm septic arthritis.

Given the uncertainty regarding cardiac risk, current guidelines from the AHA [10] and Canadian Infectious Diseases and Immunization Committee [9] recommend secondary prophylaxis with penicillin for a minimum of one year following diagnosis, with echocardiographic evaluation at diagnosis and again at the end of the prophylactic period. If no evidence of carditis is identified after one year of prophylaxis, discontinuation may be considered. This approach reflects the precautionary principle: in the absence of robust data confirming the cardiac safety of withholding prophylaxis, a time-limited course balances the risk of RHD against the burden of prolonged antibiotic exposure.

## Conclusions

ARF and PSRA represent a diagnostic challenge given their considerable clinical overlap. Clinicians should be aware that in the presence of a concurrent suppurative infection, facets of the Jones criteria such as fever and elevated acute phase reactants may be misattributed. In cases where joint cultures are negative due to pre-treatment with antibiotics, PCR testing of synovial fluid is an important adjunct to clarify the etiology of joint involvement. Given the diagnostic uncertainty inherent in these cases, a risk-averse approach to management is prudent, with adherence to established guidelines for prophylaxis and echocardiographic surveillance to minimize the risk of further complications.
